# The value of hackathons in integrated knowledge translation (iKT) research: Waterlupus

**DOI:** 10.1186/s12961-021-00785-z

**Published:** 2021-11-24

**Authors:** Francesca S. Cardwell, Susan J. Elliott, Ann E. Clarke

**Affiliations:** 1 Department of Geography & Environmental Management, University of Waterloo, Waterloo, ON Canada; 2 Division of Rheumatology, Department of Medicine, Cumming School of Medicine, University of Calgary, Calgary, AB Canada

**Keywords:** Integrated knowledge translation, Systemic lupus erythematosus, Hackathons

## Abstract

**Background:**

Despite a growing movement toward a knowledge-user-driven research process, our understanding of the generation, implementation and evaluation of specific approaches in the integrated knowledge translation (iKT) toolbox that aim to engage health and healthcare knowledge users is limited. Health hackathons offer an innovative approach with potential to generate direct and indirect health-related outcomes benefitting participants, knowledge users and the broader population. In May 2019, our research team hosted Waterlupus, a health hackathon to improve the economic lives of individuals with systemic lupus erythematosus (SLE) in Canada. Waterlupus was held with a multi-stakeholder group of 50 participants that included advocacy organization representatives, policy-makers, researchers, physicians, individuals with lived experience and students. While the hackathon generated viable solutions with the potential to positively impact the lives of individuals with SLE, understanding how participants perceived the hackathon as an iKT tool is critical in the planning and implementation of future iKT research.

**Methods:**

Semi-structured in-depth telephone interviews were conducted with Waterlupus participants (*n* = 13) between August and November 2019 to (1) explore participant experiences of the hackathon; (2) investigate participant-identified hackathon outcomes; and (3) elicit recommendations for future iKT research using health hackathons.

**Results:**

Participants provided feedback on the format and organization of Waterlupus, and identified direct and indirect outcomes to knowledge users, students and researchers beyond the innovations generated at the event. While the majority (*n* = 11) had never participated in a hackathon prior to Waterlupus, all 13 stated they would participate in future hackathons. Positive outcomes identified include connecting with students and other SLE stakeholders, the formation of professional and support networks, increased awareness of SLE, as well as the innovations generated. Participant recommendations for future health hackathons include the addition of stakeholders from industry or technology, and the need for clear and designated roles for stakeholders to ensure efficient use of resources.

**Conclusions:**

This work contributes to a limited literature regarding the use of health hackathons for social innovation, and offers knowledge-user suggestions relevant to the implementation of future iKT events, and hackathons specifically.

**Supplementary Information:**

The online version contains supplementary material available at 10.1186/s12961-021-00785-z.

## Background

### Why integrated knowledge translation?

Despite the need for evidence-based practice and policy to improve population health, effectively applying biomedical, clinical and health services research outcomes presents distinct challenges that can lead to a “gap” between knowledge and practice [1–4]. Integrated knowledge translation (iKT) is a research approach that values involving knowledge users as equal partners alongside researchers throughout the research process, not solely at the end of a study, in order to create research that is more relevant and useful to the knowledge users themselves [4]. To enhance the relevance and application of research outcomes and generate timely and useful solutions to identified health problems, iKT strategies can address the challenges that result in gaps between research and practice (e.g., outdated or ineffective use of evidence, lack of time or tools to make sense of research outcomes, differing values between stakeholders) [5–8]. While bridging the knowledge to action gap is not a novel idea [9], there is a growing movement toward a knowledge-user-driven research process in order to develop research questions and outcomes that best meet their needs [10]. In the context of public health research and practice, understanding the availability and efficiency of tools that can equip researchers, knowledge users, policy-makers and other stakeholders to make evidence-informed decisions and effectively communicate health-related messages to the wider public is critical.

Despite calls for the acceptance of iKT research, actual resources for, and evidence supporting its success, remain limited [11]. There is little research describing the generation, implementation or evaluation of specific approaches and methods in the iKT toolbox that aim to engage health and healthcare stakeholders. Health hackathons offer one possible approach with potential to generate direct and indirect innovative health-related outcomes that benefit participants, knowledge users and the broader population; indeed, hackathons are driven by iKT principles and aim to involve end-users in the coproduction of evidence-based and knowledge-user-driven solutions [7].

“Traditional” hackathons are historically associated with programming and computer science [12–15] and have been used to generate innovations whereby participants (e.g., programmers, software developers) collaborate intensely on software projects over a short period of time [16]. Hackathons have consequently emerged as an effective approach to encourage technological innovation and software development in a range of spaces (e.g., academia, open data, music) [16, 17]. At “traditional” hackathons, participants gather in teams to generate innovative solutions in a short time frame [18, 19]. While they were initially conducted as “problem-focused programming events” [20, 21] concerned with software development (indeed, the term was coined by software developers in 1999 [17]), hackathons are now conducted to cover a range of technologies [20] and to promote innovative thinking to tackle civic and ecological issues [18, 19]. These events provide opportunities for participants to collaborate and create networks that can last beyond the event itself [17], and documented tangible outcomes include the development of technical (prototypes [17, 22–24], bug fixes [22, 25], product features [22]) and non-technical artefacts (e.g., visualizations [22, 26, 27], publications [22] and other new or improved documentation [28]), as well as intangible outcomes that include learning or acquiring new skills (e.g., code camps [17, 18]) that can be applied to tackle real-world challenges [28–31], networking [19, 28] and collaboration [32, 33], entrepreneurship [19, 34, 35], and the generation of new knowledge, ideas [26, 36] and increased awareness about hackathon themes [19, 30, 37].

Traditional hackathons are typically executed with certain positivist characteristics (e.g., marathon coding sessions with informal eating and sleeping) that may reflect some participants’ preferences but can exclude others who may not feel like they belong at events structured in this way [20]. While stakeholders in attendance can vary, traditional hackathons often include students and university and company representatives [17]. Further, mentor involvement at traditional hackathons can be limited, as traditional approaches assume participants have sufficient expertise to work on projects on their own; the occasional support provided by this approach may not be sufficient for certain populations whose voice is essential for generating valued innovations (e.g., newcomers [29], other knowledge users with limited traditional hackathon experience [20]). This traditional rigid structure which involves only certain knowledge users may exclude more relevant populations [20], such as patients or other vulnerable groups with lived experience essential to problem solving. Involving a broader group of knowledge users can therefore ensure a more inclusive approach that generates meaningful innovations that include the voices of the most vulnerable.

In contrast, health hackathons first appeared in 2011 through the Massachusetts Institute of Technology (MIT) [38]. Although similar in their aim to generate innovation in a compressed period of time, health hackathons are multidisciplinary events that bring together diverse stakeholders to address complex health challenges [7, 12, 15], and have addressed a range of health issues including reproductive health, diabetes, Ebola [39], multiple sclerosis and dementia [40], and more recently COVID-19 [41]. Further, outcomes from a recent review of 12 international health hackathons [38] found that from the events reviewed, tangible outcomes of the hackathons included the generation of new project ideas, and multiple companies formed and patents filed. Further, 87% of participants surveyed stated they would attend another hackathon in the future [38].

### Waterlupus health hackathon

In May 2019, our research team partnered with the Greenhouse for Social Innovation at the University of Waterloo (see: https://uwaterloo.ca/stpauls/greenhouse) to host the Waterlupus health hackathon; to our knowledge, the first iKT activity focused on the coproduction of knowledge to improve the economic lives of individuals with systemic lupus erythematosus (SLE).

While a detailed summary of the methods and outcomes associated with Waterlupus has been published elsewhere [7], Waterlupus aimed to generate innovative, feasible and actionable solutions and leverage expertise between stakeholders with the goal of addressing the economic life needs of individuals with SLE in Canada. SLE is a chronic autoimmune disease characterized by periods of remission and relapse. SLE manifests, in its milder forms, as rash and arthritis, and in its more severe forms as life-threatening renal, cardiopulmonary and neurological involvement [42]. The disease disproportionately affects women and non-Caucasians [42], and is therefore gendered, racialized, idiosyncratic, and often episodic, unpredictable and invisible. Those with SLE experience distinct physical, emotional and social challenges (e.g., altered career trajectories, gender role definitions), and often experience a contraction of social networks and loss of identity, making those impacted especially vulnerable [43–46].

Individuals with SLE and their families experience considerable direct and indirect economic challenges [47, 48], and affected individuals often experience a less satisfying working life largely due to the invisible, idiosyncratic and episodic nature of the disease [44]. Further, pharmacological treatment options remain limited and are often poorly tolerated or ineffective [49], emphasizing the need to look to broader non-pharmacological interventions; indeed, to effect change and improve the economic lives of individuals with SLE, Waterlupus was held with a multi-stakeholder group of 50 participants (see Table 1, Appendix for Waterlupus participant breakdown), including lupus advocacy organization representatives, policy-makers, researchers, physicians, individuals with lived experience and students over a period of 28 hours at the University of Waterloo in May 2019 (see Table 2, Appendix for a schedule of the event).

**Table 1:** Interview participant overview

Participant number	Gender	Residence	Role at hackathon	Previous hackathon experience?
1	Female	Ontario	Policy mentor	N
2	Female	Alberta	Advocacy mentor	N
3	Male	Ontario	Lived experience mentor	N
4	Male	Ontario	Student	N
5	Female	Ontario	Policy mentor	N
6	Female	Ontario	Student	N
7	Female	Ontario	Advocacy mentor	N
8	Female	United States	Advocacy mentor	N
9	Male	Ontario	Student	Y
10	Male	Alberta	Lived experience mentor	Y
11	Female	Alberta	Lived experience mentor	N
12	Female	Ontario	Student	N
13	Female	British Columbia	Lived experience mentor	N

**Table 2:** Hackathon highlights

Hackathon highlight	Number of participants (%)*	Mentions (%)**
Presentations	5 (38)	7 (54)
Engaging with students	3 (23)	3 (23)
Engaging with mentors	2 (15)	3 (23)
Pitches	2 (15)	2 (15)
Learning	2 (15)	2 (15)
World Café	2 (15)	2 (15)
Meeting others	1 (8)	1 (8)
Total	13 (100)	20 (100)

*Presents number of participants mentioning variable from total sample (also as a percentage)

**Presents number of mentions of variable and percentage of total mentions

A primary criticism in hackathon research exists in that these events can often exclude vulnerable populations [50] (e.g., they are typically attended by male participants who have pre-existing experience in coding or software development). Waterlupus aimed to address this criticism; this event was female-focused as SLE is gendered and affects primarily women. Involving women with lived experience is critical to ensure the innovations generated address their unique needs. Although females are disproportionately impacted by SLE [42], we also involved two males with lived experience to ensure their voices were included. Further, while the involvement of mentors at hackathons has been documented [29], it has typically been limited to students and company and university representatives, and with the exception of the Nolte et al. (2020) hackathon [29], there are few examples that document the involvement of wider community members as mentors. At Waterlupus we aimed to address this gap by involving a broad range of knowledge users as mentors, thus ensuring as many relevant voices as possible in the generation of innovations, and thus an inclusive and accessible event [20].

To kick off Waterlupus, one mentor with lived experience spoke of how SLE has impacted her life. This introduction engaged the hackathon participants and also helped the students and other mentors gain a deeper understanding of the economic challenges related to living with SLE. The GreenHouse then facilitated a World Café, whereby student participants discussed a series of questions with the mentors in order to brainstorm ideas and identify research, policy and social innovation gaps. The World Café also acted as an ice breaker for participants, many of whom had never met prior to Waterlupus. During the World Café, questions were used to prompt discussion (e.g., What does economic quality of life mean to you?). Student participants were encouraged to discuss their interests, backgrounds and ideas, and some started to pair up or create small teams as the conversations progressed. While students were permitted to sign up as part of a team, many registered as individuals and created teams throughout the World Café. Following the World Café, the researchers and the GreenHouse team facilitated a number of unique workshops (e.g., Deep Dive into Research Workshop, Team Formation Workshop, Ideation Workshop) to help facilitate team formation and ideation beyond the World Café, and an interactive working period continued into the evening on the first day (Friday) and throughout the following day (Saturday). While the teams were not mentored directly by a single individual, all mentors were available throughout the hackathon, and students were encouraged to approach them with questions or for feedback, and the mentors were encouraged to circulate through the teams to hear about their innovations during the open working period. At the end of the second day, the student teams pitched their ideas, and three judges deliberated to identify a first-, second- and third-place team based on pre-established judging criteria (outlined elsewhere [7]).

Although the Waterlupus outcomes are broad [7], the primary innovations generated offered feasible and viable solutions with the potential to positively impact the economic lives of, and the social and economic challenges faced by, individuals with SLE (e.g., workplace barriers, social isolation, stigmatization) [7]. The winning team pitched their idea to collaborate with advocacy organizations and clothing brands to increase accessibility of sun-protective clothing suitable for different environments (e.g., employment, school), while the runners up developed an idea for a professionally moderated online social network for individuals with SLE to connect with relevant employment-related information and resources. Despite the challenges associated with COVID-19, members of both teams intend to continue progressing their innovations with collaboration with the research team and other relevant stakeholders.

To ensure the involvement of knowledge users throughout the research and make the event as inclusive as possible, we aimed to engage stakeholders at each stage of the research process including the planning, implementation and now evaluation of the hackathon; we drew on the expertise and experiences of researchers, a physician, individuals with lived experience and members of the GreenHouse team while planning the hackathon. Further, our intention was to engage as many different kinds of knowledge users as possible in the hackathon itself (e.g., policy-makers, advocacy organization representatives, individuals with lived experience, medical professionals, researchers) which resulted in the generation of relevant and useful innovations with high potential to positively impact the lives of individuals with SLE.

### iKT beyond Waterlupus: where do we go from here?

While the results of Waterlupus contribute to a limited literature regarding the use of health hackathons for social innovation, the success of Waterlupus also emphasizes the value of hackathons as an iKT tool that can be used to generate innovative ideas to address complex health challenges as part of a larger iKT process [7]. To go beyond solely documenting the results of Waterlupus, and to ensure hackathons—as an iKT tool—can enhance the relevance and application of research outcomes, we believe it is critical to understand how hackathon participants (e.g., knowledge users) perceive the value of hackathons, and explore how future iKT science, and hackathons specifically, can best meet their needs. This study therefore aims to involve knowledge users as equal partners at each stage of the research process (e.g., in the planning, execution and evaluation of Waterlupus), and more specifically aims to (1) explore Waterlupus participants’ experiences of the hackathon; (2) investigate participant-identified outcomes of the hackathon; and (3) elicit recommendations for future iKT research using health hackathons.

## Methods

This manuscript presents results from qualitative semi-structured in-depth interviews on participant experiences and perspectives of the Waterlupus health hackathon. The Waterlupus health hackathon and subsequent follow-up interviews employed an iKT approach whereby members of the SLE community (knowledge users; individuals with lived experience, researchers, lupus specialists, advocacy representatives, health policy-makers) were involved as partners throughout the research, and not solely in the consumption of results [4]. In this vein, the work was developed both *for* and *with* the SLE community, not just “on” or “about” patients diagnosed with SLE [44]. This approach values knowledge users as partners throughout the research process, not solely as an end-of-study point for consumption of the research findings [10, 51], and contributes to the development of long-term partnerships and shared understandings between scientists and knowledge users [8]. While the broad focus of the Waterlupus hackathon was to understand the economic experiences of individuals with SLE and develop non-pharmacological interventions to improve the working lives of these individuals, the inclusion of other knowledge users in both the hackathon and the follow-up interviews helps to contextualize the challenges of individuals with SLE in different regions of North America, and provides an opportunity for different knowledge users to share their perspectives of the hackathon and provide suggestions that may be relevant for their user group’s involvement in future iKT health innovation events.

At the end of Waterlupus, all participants (with the exception of members of the research team) were asked if they were interested in learning about future iKT research (see Table 1, Appendix for hackathon participant breakdown). All participants who expressed interest (*n* = 27) were contacted by email in July 2019 and invited to participate in a semi-structured in-depth follow-up interview to increase understanding of their experiences and perspectives of the hackathon. All participants that responded to the invitation email (*n* = 13) participated in the follow-up interviews (48.1% response rate). To meet inclusion criteria, participants were 18+ years of age, and participated in the entirety of the Waterlupus weekend. To ensure maximum variation of participant characteristics in our sampling approach, we aimed to ensure participants were representative of the varying demographics, regions of residence, hackathon roles (e.g., students, lived experience, policy and advocacy mentors) and previous hackathon experiences of Waterlupus participants.

The interview guide (see Interview Guide in the Appendix) was developed by the authors following Waterlupus. All research team members were present at the hackathon, and observational notes from the hackathon were used in combination with questions believed relevant to the research objectives (for example, to assess participants’ experiences of the hackathon, perceptions of the hackathon outcomes, and recommendations for future iKT research) to guide the development of the interview guide. The guide was developed by the first author with input from the other members of the research team, and pilot tested before the start of the interviews; this peer debriefing step contributes to the credibility of the qualitative data collection process (see Baxter and Eyles [52], especially Table 2).

Prior to recruitment, this study received ethics approval from the [University of Waterloo] Research Ethics Board. Participants (*n* = 13) were interviewed by telephone, and interviews lasted approximately 30 min. Interviews were conducted one at a time by the first author between August and November 2019 in order to gain an in-depth understanding of the experiences of each participant and understand how their role may have impacted their experience at the hackathon. All participants provided verbal consent for their interview to be audio recorded, and recordings were subsequently transcribed verbatim. Throughout the interviews, member checking was ongoing to ensure the participant responses were correctly interpreted.

All interviews were undertaken, transcribed and proofed by the first author. Following accepted qualitative analysis protocols [53], an initial theme code was developed and sorted into themes and subthemes. Themes were generated both deductively (themes for inclusion in the codebook were developed based on the research objectives and interview guide) and inductively (e.g., unanticipated themes that emerged throughout the interviews and coding process). The initial code set was then used for a detailed review of two randomly selected transcripts which were then coded to pilot test the theme code set. The first and second author then reviewed the transcripts and codes and noted any discrepancies for resolution [54]. The authors revised the theme code set, and it was then used to code all transcripts using line by line coding assisted by NVivo for Mac. To enhance consistency and credibility in the thematic analysis, the first author met with the second author regularly throughout the coding and analysis process to discuss preliminary findings, while the third author contributed to the interpretation of results.

At the time of interview, 10 of the 13 participants identified as female, and the majority (*n* = 8) of participants were residents of Ontario, three from Alberta, one from British Columbia and one from the United States (see Table 1 for participant overview). All members of the research team attended the hackathon and so had interacted with all participants at this event. See Additional file 1 for the completed Standards for Reporting Qualitative Research (SRQR) checklist.

## Results

Results are presented around participant experiences and perceptions of the Waterlupus health hackathon and recommendations for future hackathon or iKT events. To provide a comprehensive summary of the interview themes, we present tables that summarize the relative importance of the themes that emerged throughout the interviews, complemented with participant quotations throughout.

### Hackathon experience

To establish rapport, interviews began with a discussion of participants’ experiences of the hackathon and with SLE. With respect to hackathon participation, four interviewees attended as student participants, while three attended representing advocacy organizations, four attended in the role of mentors with lived experience, and two attended as policy mentors. When asked about their experiences and familiarity with SLE prior to Waterlupus, three participants (all attending as mentors with lived experience) described a personal SLE diagnosis, while three reported being familiar with SLE through their employment (the advocacy mentors). Three participants had a family member or friend impacted by SLE (one participant with lived experience, one policy mentor, one student), and four (three student participants, one policy mentor) identified having no prior experience with SLE. The majority (*n* = 11) had never participated in a hackathon prior to Waterlupus, while two had participated in a technological hackathon, but not related to social innovation or health.

Participants were then asked about their experiences at the hackathon. Respondents discussed how they perceived their role at the event; seven (primarily the advocacy, policy and lived experience mentors) believed they were in attendance to support and provide input to student participation. Two mentors with lived experience described the value of sharing their lived experiences with other attendees, while five participants spoke of attending the hackathon in order to learn more about the lived experiences of those with SLE. The mentors with lived experience not only offered their expertise on the economic challenges of living with SLE, but discussing these experiences provided an opportunity to not only share how their illness impacts their daily lives, but also increase awareness of SLE more broadly. This sentiment was shared by both attendees and mentors, as participants spoke of the challenges of describing the complexities and nuances of the illness to friends, family, employees and community members when diagnosis occurs. As one lived experience mentor identified:

For these people going forward with the information they got at Waterlupus, they may be or have a relative, or friend or coworker or employee who shares with them that they have a diagnosis of lupus, and it will be much easier for the patient then to perhaps speak to someone who knows about the disease. (Interview 13)

More specifically, the generation of innovations was identified as valuable by three participants; indeed, the primary aim of the hackathon was to develop non-pharmacological interventions to improve the economic lives of individuals with SLE in Canada, and at the end of the weekend there were two winning innovations out of five pitches. The advocacy, policy and lived experience mentors spoke extremely highly of the student participants, and were both impressed and thankful for their participation; indeed, although these mentors described their primary role was to share their expertise and provide feedback to the students, they also spoke of learning and being energized from the students and their interactions with other attendees:

I considered my role to be very much of learning and being there to support the students and advise them and give them recommendations, but also take on a learning role. I was able to learn from the students. (Interview 8)

I found the hackathon experience very invigorating… there was so much energy, and curiosity and interest in the room by these individuals, that it felt inspiring and that anything was possible. It really did feel like anything was possible. (Interview 1)

When discussing their hackathon experiences, the majority (*n* = 11) spoke positively about the event. Eight individuals described the value of meeting and interacting with other hackathon participants; indeed, all but one participant described keeping in touch with others from the hackathon socially (*n* = 6) and professionally (*n* = 5) in the time since Waterlupus. For example, participants—especially those working or volunteering with SLE advocacy organizations—felt that the hackathon provided an opportunity for them to connect with other stakeholders in the SLE community who are living/working in different geographical locations (e.g., in Ontario, Alberta, or the United States):

It was an awesome experience. It was great because I got to connect with the lupus society out there… we had not connected with them prior to the hackathon, so that was a very positive experience, and moving forward we have more of a relationship, and so that’s definitely something we want to carry forward… that was a great opportunity that you provided that we got to connect, I got to meet board members, talk to them one on one, and develop friendships with them. (Interview 2)

The educational value of the hackathon for both student and mentor participants was also identified (*n* = 5) by various groups of knowledge users, particularly as most of the students had no prior experience with SLE:

I thought it was really positive. It was really interesting for me to see these students come together, and a lot of them weren’t even familiar with lupus until the past couple of weeks... So I thought it was fascinating how quickly they picked up information and they were able to learn what the problems were and come up with solutions, and I was most impressed with how quickly they were able to do that. It just impressed me a lot that they were able to come up with such good ideas in such a short time frame without much background on the disease. (Interview 8*)*

Hearing the student team pitches (*n* = 3), participating in the World Café (*n* = 2) and observing the research presentations (*n* = 1) were also described positively (see Table 2, Appendix for Waterlupus schedule).

Participants were then asked about what they considered to be the highlight of Waterlupus (Table 2). Five respondents identified the presentations by the researchers and individuals with lived experience, as they provided context and allowed participants to feel engaged with the goals of the event:

My favourite part was really hearing the stories of people who had lupus at the very beginning. I really enjoyed that quite a bit…You know, just the speakers at the beginning and the stories and how it affected them. It kind of made it much more personal, I felt more personally interested. And how people are living, and just their stories opened my eyes, like how severely this can impact people’s lives. (Interview 10)

The student participants reported feeling that the presentations were especially valuable, as the research presentations were their first introduction to the economic challenges associated with living with SLE:

All of those talks and the structure gave us a head start for understanding lupus, because there’s only so much that reading can help me do, because what we got from the first day is more about current research and what is being done, and all of the talks focused on what has already been done, so that helped generate ideas on what else can be done. (Interview 9)

The World Café occurred immediately following the research presentations, and aimed to both provide an opportunity for the lived experience mentors to continue to share their perspectives, and also allow the students and other knowledge users to familiarize themselves with the problem space. While two individuals (one policy mentor who expressed an interest in using this type of event in future work, and one student participant) mentioned the World Café as a highlight, these individuals were extremely enthusiastic about the role this event played at Waterlupus:

The brainstorming session (World Café) got me thinking more about the problem space and how lupus patients might be affected, so that was pretty cool! That was something that was really unique and that stood out to me. (Interview 12)

The World Café! I thought the World Café was wonderful. Even the music, just added some fun to it… (Interview 1)

Participants also recognized the value of engaging with the students, and described the quality of their pitches after only a short working period. The lived experience mentors in particular voiced how impressed they were with the students, and also felt enthusiastic that this group of individuals with no prior knowledge of the illness were so keen to engage with SLE:

I really enjoyed the students, and I thought the pitches that they did were excellent. I was very impressed by the ones who won… I think to go away knowing that all of these young, academic people in different disciplines knew more about lupus at the end than they did, and as they go forward in their careers, they would have that knowledge of the issues that people with lupus face. And I think there’s just exponential potential coming out of having educated these young people. (Interview 13)

Participants were then asked to discuss what they perceived to be the primary outcomes of the hackathon. A number of positive outcomes were identified (Table 3), including the engagement of students at the event (*n* = 7; described by the lived experience, advocacy and policy mentors), the innovations generated (*n* = 7), the professional networking and capacity-building that occurred (*n* = 5; primarily described by the policy and advocacy mentors), and increased awareness of SLE amongst participants (*n* = 4; primarily identified and especially valued by mentors with the advocacy and lived experience mentors). Indeed, all 13 participants stated they would participate in a future hackathon.

**Table 3:** Perceived hackathon outcomes

Hackathon outcomes	Number of participants (% of total)*	Mentions (% of the total)**
Student engagement	7 (54)	8 (62)
Innovations	7 (54)	7 (54)
Professional networks/capacity-building	5 (38)	5 (38)
Increased awareness of SLE	4 (31)	4 (31)
Engaged with new people	3 (23)	4 (31)
Support networks generated	2 (15)	3 (23)
Mentors	2 (15)	2 (15)
Individual learning	1 (8)	1 (8)
Total	13 (100)	34 (100)

*Presents number of participants mentioning variable from total sample (also as a percentage)

**Presents number of mentions of variable and percentage of total mentions

The two most frequently identified perceived outcomes are thematically similar: the speed and quality of the innovations generated in the tight timeline (*n* = 7), and the engagement of the students involved in creating those innovations (*n* = 7), in collaboration and with support from the mentors:

I think one of the most valuable aspects was being able to quickly pinpoint an aspect of the issue at hand, that would bring the greatest improvement in quality of life, given the investment in the solution. So, we had to get to that point very quickly…I think often times people go in with the intention of wanting to help, and the steps and the pace are so slow and the individuals who would benefit are just watching from the sidelines suffering, essentially, and just watching a very, very slow-moving process. But the hackathon enables you to get almost a quick win, gain some momentum, find some trust and some hope for the individuals with the disease, and build some positive momentum that can carry you as you go through a longer-term process. (Interview 1)

While the lived experience, policy and advocacy mentors were extremely vocal in their praise of the students and the innovations, the student participants also spoke positively of the support they received from the mentors present, and how this shaped the development of their ideas and innovations:

We got to talk to the patients with lupus and the researchers from my university to check our ideas and give their inputs and they were so honest… I think it was the second day of the hackathon. That was probably the most influential in helping me and my teammates realize what’s a realistic idea and what’s not… so the fact that every mentor was prepared to talk to everyone was really a highlight and I did not expect it, but it was really good. (Interview 9)

The professional and support networks that were generated and enhanced were identified as a primary outcome by five and two participants, respectively. In this context, engaging with the knowledge users themselves through the research process was identified as something that, despite its value, seldom happens in public health—particularly with respect to including individuals with lived experience themselves. Not only was this beneficial in strengthening relationships and networking at Waterlupus, but the inclusion of a diverse group of knowledge users in hackathons—and Waterlupus specifically—can directly improve the iKT experience, strengthen stakeholder relationships, and consequently enhance research outcomes:

I think in general for me, hackathons are like a really good networking session, and I get to meet new people, I get to connect with people I’ve never seen before, that was nice. (Interview 13)

I also think just having that time to chat with people about their experiences was majorly beneficial. So, you know, for patients to work through some of their experiences, and to talk to people who aren’t patients but who want to learn about their experiences, the experiences of people who have lupus, just having that dialogue I think is important. You know, I’ve been in a lot of meetings, but I haven’t been in a lot of meetings with the actual intervention group I guess you could say in public health or health research, so having that opportunity to talk to the group you are directly impacting—we don’t get that opportunity a lot so you’re kind of always guessing about things, about evaluation and making recommendations about the impact of programmes you are sort of guessing, so having those groups right at the table from the start I think made this event and process I think super valuable. (Interview 5)

Participants also identified the learning that occurred, and spoke of the awareness generated at the event (*n* = 4) as a primary outcome. With respect to the educational component specifically, seven participants discussed the intense learning by the students attending Waterlupus, many of whom knew little about SLE in advance. Other long-term skills obtained (e.g., critical thinking, communication, liaising with various stakeholders) were also described by the students and other knowledge users (e.g., this policy-maker):

I think long-term for the individuals who participate in a hackathon, you’re building a skill for life, having these experiences as short time-wise as they are, they’re so intense, I’m absolutely certain that they leave something with you, and the next time you are faced with an issue you’re more likely to start to “ok, so what is the key underlying issue, how can, what are the other ways we can look at this?” and there, I would think it puts you in a position where you want to work with people who have differing opinions than you, different perspectives, and we know that’s the crux of innovation, that’s the crux of changing the way we see and think about things. So while it’s small, the hackathon for those involved in it does have a lasting impact. (Interview 1)

To investigate participants’ previous understanding of hackathons, we asked about their expectations of Waterlupus prior to attending. Despite the positive feedback following the event, advance knowledge of hackathons was limited. Six participants stated they knew little about hackathons and had no specific expectations, while two had never heard of the term prior to being invited to Waterlupus:

I was still marvelling at the fact that this was called a hackathon, I had no idea what I was walking into and I thought that was good. You know, initially I thought, oh, what could my value add possibly be? But I trusted that the person that invited me knew the compilation of who she was putting in the room, and who the researchers were putting together, and I trusted in that and I showed up. (Interview 1*)*

Despite knowing little about hackathons, four participants described their excitement to attend, specifically related to engaging with new people at the event:

I was really excited about meeting new people and hearing what they had to say and what their innovative idea is. And at the same time I was also aware that as it was a team event I would be able to meet other people and work with them, so that’s what I was most excited about to meet new people and to listen to what they have to say and what they have done. (Interview 4)

Two participants had never heard the term “hackathon”, and one student assumed that Waterlupus would be a technological hackathon when they registered due to their previous understanding of the term. This emphasizes the need to elaborate around the objectives of the hackathon in recruitment materials when organizing nontraditional social or health hackathons.

### Future recommendations

While most participants spoke favourably of their experiences at Waterlupus, participants were asked to provide feedback and suggestions for future SLE-related research, and offered recommendations for future iKT research (e.g., future hackathons) more broadly. The majority of both the Waterlupus attendees and the interview participants had never previously attended a hackathon (social, technological or health-related), so feedback was based purely on their experiences at Waterlupus and was generally not reported relative to previous hackathon experience. With respect to the types of stakeholders perceived to be necessary attendants at health hackathons, participants believed that the policy, patient, medical professional, advocacy and research mentors at Waterlupus provided a diverse variety of important voices to contribute to the stated aims of the hackathon. Some participants stated explicitly that they thought the stakeholders in attendance were sufficient, while the need for industry (e.g., from healthcare) representatives (*n* = 4) and experts in technology (*n* = 1) for those interested in technological intervention support were also identified by student and lived experience mentors:

Maybe someone from a company, so a true innovator, someone you know that they built a successful company, just to give the students that entrepreneurial experience. (Interview 2)

While a rheumatologist was a valuable Waterlupus attendee, participants discussed the merits of including additional medical professionals (e.g., nurse, nutritionist) as resources. Participants with lived experience explained that they thought this would be beneficial and provide a more diverse range of perspectives, as patient experiences of the healthcare system are diverse and include a variety of healthcare providers. Although challenging, one advocacy representative also described the need to engage individuals with lived experience who may not have heard about the event, may not have internet access or may be unable to participate in employment or voluntary work due to their SLE; indeed, the perspectives of those not in attendance would contribute to this study and strengthen the innovations generated, and are critical in future iKT research more broadly.

Similarly, two participants spoke of the need for more geographic representation from the mentors. For example, one participant with lived experience identified the value in having representation and perspectives from across Canada to increase understanding of the experience of SLE in additional provinces:

The experience of treatments varies from province to province, so some provinces have more challenges to access to specialists and access to medications, so being a lupus patient in Canada is not a universal experience at all. People from different provinces may have been able to give another perspective. (Interview 13)

While we included stakeholders from three Canadian provinces and one from the United States, patients’ experiences of SLE will certainly vary across geographical contexts. Ensuring interventions that improve the economic lives of individuals with lupus and other chronic conditions are geographically and culturally suitable through feedback from patients from different geographical contexts is critical to their success.

With respect to the role of mentors, participants (*n* = 4) also suggested that the mentors and students should be provided very clear and designated roles at the event. For example, one lived experience mentor described the need for more structure with respect to how students and mentors engaged with one another, in order to maximize the time and voices of the mentors. This is especially important in the case of mentors with lived experience, who are not only managing their illness but volunteering their time at this event:

A more formalized structure, where we are working and interacting with the students more. The students were absolutely encouraged to come and see us, we were absolutely encouraged to go and see the students, and there was some of that happening. But I felt that the students were so under the gun in terms of timelines where they were so focused on getting pen to paper. So I felt like there was less focus on tapping into the human resources that were there, and they were more concerned with taking a few ideas, getting it down, getting a presentation done. I know it’s a time-sensitive thing, but I felt like the mentors with lived experience could have been better utilized. (Interview 3)

To ensure productivity and encourage innovative ideas, the need for a dynamic working environment that implements the tenants of design thinking methodology was also identified by one participant. This individual attended as a policy mentor, and brought iKT and public health policy expertise to the hackathon. For example, the need to ensure physical movement throughout the day was described:

One of the central tenants of design thinking theory methodology for innovative ideas, is that moving your body in and of itself is a great tool to get yourself to think of something from a different perspective. So you’re physically moving, so you’re going to see it from over here in the room and over there in the room, but there is a psychological shift that happens when you’re able to be up and about. Doing something interactive in a moving way can also bring people together in a way in which, if they’re not effective communicators in one way maybe they are in a different way—maybe artistically. You know, just having other tools around to allow the students to contribute in whichever way they feel most comfortable. (Interview 1)

Finally, participants were asked to identify what they perceived to be the next steps to progress the outcomes of Waterlupus (Table 4). Four participants described their enthusiasm and interest in ensuring the innovations continue to progress, and three (one student, one lived experience mentor and one advocacy mentor) discussed their continued involvement in the innovation process. For example, in addition to the enthusiasm from the lived experience mentors and the students on the innovation teams, an advocacy organization representative discussed how they are continuing to provide input to see if the innovations can be disseminated through their organization:

It’ll be great to see the model they come up with, and see if that’s something our organization would want to think about pursuing in the future. (Interview 7)

**Table 4:** Waterlupus next steps

Waterlupus next steps	Number of participants (% of total)*	Mentions (% of the total)**
Innovations to progress	4 (31)	4 (31)
Personal continued involvement with the work	3 (23)	4 (31)
Other (e.g., engagement plan, infographics)	3 (23)	3 (23)
Innovation updates	3 (23)	3 (23)
Future hackathon/other events	2 (15)	3 (23)
Do not know	2 (15)	2 (15)
Total	13 (100)	19 (100)

*Presents number of participants mentioning variable from total sample (also as a percentage)

**Presents number of mentions of variable, and percentage of total mentions

With respect to knowledge dissemination, participants also spoke of the need to continue the momentum from the hackathon. Suggestions for ongoing communication with hackathon participants and broader stakeholders include creating a long-term engagement plan to ensure the relationships generated at the event are sustained, while another participant suggested creating interactive resources to share with other interested stakeholders:

My only idea would be maybe creating a video from the weekend event to share with I don’t know, maybe potential funders, or getting students interested, or just to have on your website. tabl(Interview 2)

To build on the innovations and knowledge generated, we also asked participants to provide suggestions for future research to improve the economic lives of individuals with SLE (Table 5). While most were able to provide insight into future research priorities, one student was unsure of what future SLE research should be conducted.

**Table 5:** Future research priorities

Future SLE research priorities	Number of participants (% of total)*	Mentions (% of the total)**
Increase awareness of SLE	4 (31)	4 (31)
Other (e.g., gender lens, comorbidities)	3 (23)	5 (38)
Improving financial security	3 (23)	3 (23)
Further innovation needed	3 (23)	2 (23)
Pharmacological interventions	2 (15)	2 (15)
Don’t know	1 (8)	1 (8)
Total	13 (100)	17 (100)

*Presents number of participants mentioning variable from total sample (also as a percentage)

**Presents number of mentions of variable, and percentage of total mentions

Increasing awareness of SLE beyond hackathon participants was most frequently identified (*n* = 4) by advocacy and lived experience mentors; although participants described the need for increased awareness amongst the general population, ensuring employers and workplaces are aware of the needs of individuals with chronic illness was described as critical:

My [family member with lupus] takes a lot of time off work because she’s sometimes very unwell, and so I think having some protocols in place or information available for employers on various chronic diseases including lupus would be really useful, so people aren’t having to suffer more if they’re unwell and off work, and that they’re supported in their workplace to do that and to get better and then come back to work, that was one thing that really struck me as a really important policy issue. (Interview 5)

Consistent with the hackathon theme of improving the economic lives of individuals with SLE, improving financial security for those impacted was also described by three participants:

So pain management is one thing, but in terms of the hackathon, the financial security piece. I think partly as I was saying is for people who don’t have access to any resources and ODSP [Ontario Disability Support Program] is, you know, so difficult to live off of, and people who may not have great literacy skills or who may not be proficient in English, you know, people who are really, really struggling just to make it day to day I think sort of looking at what are options or opportunities for them as well. (Interview 7)

Other identified priorities include the need to understand the lived experiences of individuals who experience lupus as well as other chronic illnesses, and how gender impacts the nuanced experiences of individuals with SLE. While Waterlupus aimed to improve the economic lives of individuals affected by SLE, these responses emphasize the need for broader systemic change beyond the capacity of a single weekend hackathon. Although participant responses clearly identify the value of increased awareness of the economic challenges individuals with SLE face throughout the weekend, carrying this awareness forward through broader student, advocacy and policy networks is critical for real change.

## Discussion

This study reports results of semi-structured in-depth interviews with 13 participants (students, knowledge users [individuals with lived experience, policy and advocacy representatives]) of the Waterlupus health hackathon on their perceptions of the value of hackathons in iKT research. While there is a growing literature on how hackathons are useful research tools in health [30, 38], software development [17] and technological innovation [12, 14], our knowledge of how hackathon participants perceive their involvement and their perceptions of the value of hackathons as an iKT tool is limited. In this study, participants provided feedback on the format, organization and outcomes of Waterlupus, and offered suggestions relevant for planning future iKT events to ensure outcomes (e.g., the coproduction of knowledge, innovation, tools and policies) are relevant and impactful for various knowledge user groups.

A recent systematic review of outcomes in hackathon research identified both tangible (e.g., technical and nontechnical artefacts such as new prototypes, innovations and publications) and intangible (e.g., ideas, networking, learning and increased awareness about the hackathon theme) outcomes [19] that can come from (primarily technological) hackathons. Our interview results reiterate the importance of these tangible and intangible outcomes, as interview participants heavily emphasized the networking, interdisciplinary collaboration and increased awareness of SLE from the event.

Further, in their discussion of iKT in the context of a large food allergy research programme, Dixon and Elliott [8] identify the benefits of measuring research outcomes beyond the knowledge gaps filled in any one research project. For example, research that increases knowledge about the nature of science and iKT, increases lay awareness of public health problems, elicits cultural change, builds partnerships between stakeholders, and improves additional skills (e.g., communication, dissemination), contributes to future research, science and policy in meaningful ways. Consistent with the identified advantages of iKT research design by Dixon and Elliott [8] and both the tangible and intangible hackathon outcomes identified by Angarita and Nolte [19], participants in this study discussed not only the innovative outcomes generated at Waterlupus [7], but also described some of the indirect and longer-term outcomes of participation. For example, the professional networks generated pave the way for future collaboration; indeed, the research team continues to partner with students, mentors with lived experience, and advocacy organizations on the innovations as well as additional SLE-related research. Further, the need to rethink how we measure and value research outcomes [8] is emphasized by the indirect positive outcomes identified by participants; that is, the increased awareness of SLE amongst students, the development of other skills (e.g., communication, presentation, critical thinking) for both the student and knowledge user participants, and the support networks generated between mentors with lived experience. Although less quantifiable, the “two-way learning” associated with hackathon participation (e.g., mutually beneficial interactions between researchers and knowledge users) [55] was described by various groups of interview participants (e.g., student and mentor participants alike), and emphasizes the value of hackathon participation for all participants beyond the immediate research outcomes.

While concerns of research underutilization are often considered a dissemination failure, research questions and objectives that do not address knowledge user-identified problems are suggested to contribute to the knowledge–action gap [11]. This suggestion emphasizes the need to involve all relevant knowledge users throughout the research process; to ask the right questions, generate solutions and disseminate results that will meaningfully change policy and practice. Previous literature has documented that traditional hackathons involve mentors including students and university and company representatives [17], and a primary criticism in hackathon research exists in that these events are often male-dominated, do not involve groups with limited hackathon experience, and can often exclude vulnerable populations [50]. Waterlupus aimed to address this criticism and involved a broad group of stakeholders, including individuals with lived experience of a gendered illness affecting primarily women, most of whom had no previous experience attending a hackathon. Interview participants, including students and other mentors, spoke of the benefits of involving mentors with lived experience at Waterlupus, emphasizing the need to include not only researchers, policy-makers and industry representatives, but the individuals directly impacted by the issue at hand*.*

Further, despite the potential to generate greater societal impact using an iKT approach, one of the primary challenges of iKT more broadly includes the time and resources required to effectively involve multiple knowledge users with the same end goals [3]. Hackathons are short and efficient processes [17], in which a range of stakeholders are encouraged to engage over the course of a short time period [7, 16]. The “easy win”, as identified by one of the participants, associated with participation in a hackathon can address the temporal and other resource-related challenges associated with iKT research [3].

Although the variety of knowledge user voices in attendance (individuals with lived experience, advocacy and policy representatives, rheumatologists and researchers) was described by participants as one of the valuable contributions of the hackathon, ensuring the knowledge users in attendance are utilized as effectively as possible is critical. In the context of policy-makers and healthcare professionals, there are challenges associated with engaging individuals from different geographic locations for the duration of a weekend due to work, familial and other engagements. With respect to individuals with lived experience, many were balancing employment, voluntary work, family responsibilities and the management of their disease. Waterlupus participants suggested providing more structured guidelines to the mentors to ensure their time is used efficiently. Ensuring mentors feel valued and are applied strategically may also encourage participation throughout the longer-term research process. Events like the World Café and the presentations by the researchers and mentors with lived experience are especially valuable in this context. While these events filled most of the evening on the first day of the hackathon and involved a significant amount of participation from all attendees, both student and mentor participants spoke highly of each, and interview responses highlighted their contributions to increasing awareness of SLE amongst the hackathon participants.

Participants also discussed the need to include additional stakeholders to support the student teams: healthcare professionals, technological experts and industry representatives were all identified. While engaging individuals beyond those directly impacted by the research problem can be challenging [56], applying their expertise to develop knowledge user-generated solutions that are relevant across stakeholder groups is important for future events. Further, as identified by an advocacy mentor, ensuring vulnerable populations—those whose voices are often excluded—are included is critical to develop innovations that are valued by all stakeholders, and also further contributes to ensuring inclusive hackathon research [50]. An additional challenge also comes following the hackathon, with respect to maintaining momentum and ensuring knowledge users and other participants continue with their innovation beyond the event. For example, even with the greatest of intentions from all stakeholders involved, in the context of limited resources and time constraints, ensuring long-term participation from policy-makers, healthcare stakeholders and advocacy representatives is challenging. Further, while the student participants themselves identified the benefits of hackathon participation and the winning teams have continued to work on their innovations, other competing priorities exist. Even when provided social, educational and financial resources, the student teams are balancing education (undergraduate and postgraduate), part-time employment, working on their innovations, and family responsibilities. In the context of the COVID-19 pandemic, continued involvement in the innovation development has been especially difficult; students that were working in teams have now returned to their hometowns to work remotely, some in different time zones. Despite this, their commitment remains, and the research team is committed to supporting the teams as they progress.

This study has limitations. With respect to the hackathon itself, and as identified by one of the advocacy representatives, the perspectives of the most vulnerable individuals with SLE are missing. While individuals with lived experience attended the hackathon, those who participated live in urban regions of Ontario and Alberta, participate in advocacy associations, and were supported financially to attend. Although challenging, recruiting from populations that are socially, economically or physically remote, or are most impacted economically is important to ensure all voices are included in solution design [57]. Recruiting from “hidden” populations, removing accessibility barriers and involving these individuals in all stages of the research process [57] is therefore an important future consideration for our work.

Further, the use of the term “hackathon” was identified as possibly misleading and could have presented a barrier to participation. Typically, hackathons are associated with technological events [7, 12–14]. Some participants had never heard the term prior to Waterlupus, and one acknowledged that they did not realize the event was related to social health innovation and that they believed they were attending a technological hackathon. Ensuring marketing of the event emphasizes the purpose of social innovation beyond traditional technological hackathons is important to attract a broader selection of students and knowledge users.

An additional limitation includes participant selection bias. While we included at least one participant from each group of knowledge users and aimed to interview for depth rather than to generalize, this work is limited in that the hackathon could be considered small (*n* = 50; 26% of hackathon participants participated in the interviews), and we did not include any medical professionals in our interviews (the rheumatologist in attendance at the hackathon is part of our research team). Eliciting the perspectives from a broader population of hackathon participants (e.g., from other types of hackathons including software development or those focusing on other health outcomes) or iKT research participants in general could be a valuable next step to increase our understanding of how to enhance iKT in health research and beyond.

In addition, to further reduce bias in our analysis and interpretation of results, we integrated measures to ensure rigour (52) in the interview analysis process. While the first author was the primary coder, additional members of the research team reviewed the theme code set and a random sample of transcripts. In addition, member checking occurred throughout the interviews, as the first author discussed responses with the interview participants to ensure accurate comprehension of the data. Additional interviews with a broader group of hackathon participants will help triangulate these findings, and is an important next step to understand how different aspects of health and technological hackathons can contribute to future innovative iKT research.

## Conclusions

Moving forward, it is evident that broad and innovative methodological approaches to iKT research are critical to ensure a wide variety of stakeholders are involved, their voices valued, and challenges with respect to time and priorities addressed. Not only has this study documented the direct and indirect outcomes of the Waterlupus health hackathon, but provides hackathon participant-identified feedback and suggestions for future hackathons and iKT research more broadly. Despite identified challenges, Waterlupus participants spoke enthusiastically of the direct and indirect positive outcomes, emphasizing the benefits of hackathons in iKT research. As these tools and strategies are continually developed, implemented and evaluated, the benefits of hackathons should be considered beyond technological innovation as they offer opportunities to engage knowledge users throughout the research process, generate momentum and timely innovations and can be applied with limited resources to a broad range of public health challenges.

## Supplementary Information

**Additional file 1.** Standards for reporting qualitative research checklist.

## Data Availability

The dataset supporting the conclusions of this article is not publicly available due to privacy or ethical restrictions.

## Abbreviations

iKT: Integrated knowledge translation
MIT: Massachusetts Institute of Technology
SLE: Systemic lupus erythematosus
