# Supplementary material for: The value of hackathons in integrated knowledge translation (iKT) research: Waterlupus
Source: Health Res Policy Syst. 2021 Nov 24;19:138. doi: 10.1186/s12961-021-00785-z (PMC8611392; doi:10.1186/s12961-021-00785-z)
Supplement: Supplementary file 1 — Additional file 1. Standards for reporting qualitative research checklist. [file 12961_2021_785_MOESM1_ESM.docx]

Additional File 1 – Standards for Reporting Qualitative Research Checklist

| **Topic** | **Item Addressed** |
| --- | --- |
| Title | Concise description of the study provided |
| Abstract | Summary of the study using the subheadings Background, Methods, Results, Conclusions included |
| Introduction – Problem formulation | Description of the problem and relevant empirical work provided |
| Introduction – Purpose or research question | Research objectives specified |
| Methods – Qualitative approach | Rationale for research and analysis approach described |
| Methods – Research characteristics and reflexivity | Interviewer relationship with participants acknowledged |
| Methods – Context | Setting and contextual factors (e.g., hackathon rationale and participation) identified |
| Methods – Sampling strategy | Sampling strategy described (e.g., *Waterlupus* participants) |
| Methods – Ethical issues | Approval from the University of Waterloo Research Ethics Committee statement included |
| Methods – Data collection methods | Description of start and stop dates, data collection and analysis process described |
| Methods - Units of study | Participant breakdown described in Methods section |
| Methods - Data processing | Transcription and coding process identified |
| Methods - Data analysis | Inductive and deductive theme development specified |
| Methods - Techniques to enhance trustworthiness | Member checking identified |
| Results/findings – Synthesis and interpretation | Main findings and themes presented – themes are summarized and quotes provided |
| Results/findings - Links to empirical data | Quotes included to substantiate findings |
| Discussion – Integration with prior work, implications, transferability, and contribution(s) to the field | Integration with prior work, implications and contributions are included, as well as a short summary of the main findings |
| Discussion – Limitations | Limitations to the study are identified |
| Other – Conflicts of interest | Conflict of interest statement provided in Declarations |
| Other - Funding | Funding statement included in Declarations |
